# Imbalance hepatic metabolism homeostasis in the F1 generation of endometrial *DNMT3B* conditional knockout female mice

**DOI:** 10.3389/fphys.2022.1042449

**Published:** 2022-11-11

**Authors:** Weike Li, Rufei Gao, Yubin Ding, Xuemei Chen, Xueqing Liu, Junlin He, Fangfang Li, Jing Long, Siyu Lu, Chengshun Yang, Yingxiong Wang

**Affiliations:** ^1^ Laboratory of Reproductive Biology, School of Public Health, Chongqing Medical University, Chongqing, China; ^2^ Joint International Research Laboratory of Reproduction and Development, Chongqing Medical University, Chongqing, China

**Keywords:** DNMT3B, F1 generation, liver, glucose, lipid metabolism

## Abstract

Numerous studies have suggested the possibility of explaining the etiology of metabolic syndrome through DNA methylation. DNA methyltransferase 3B (DNMT3B) plays an important role in *de novo* DNA methylation. There was an alteration in maternal (F0) endometrial function, which might lead to growth and developmental disorder in offspring (F1). In this study, we investigated the effect of maternal endometrial *DNMT3B* deficiency on the metabolism in offspring. We constructed endometrial *DNMT3B* conditional knockout female mice (cKO) which were mated with normal C57BL/6 male mice to obtain the F1 generation. Further, to study the development of these offspring, we observed them at three different life stages which included the 6-week-old juvenile, 9-week-old sub-adult and 12-week-old adult. Follow the detection of a range of metabolism-related indicators, we found that in the cKO F1 generation, liver triglyceride level was significantly elevated in 9-week-old female mice, lipid droplet deposition was significantly increased in 9-week-old and 12-week-old mice, and the expression of lipid metabolism key factors in the liver was markedly decreased except of 6-week-old male mice. These results indicate that maternal endometrial *DNMT3B* conditional knockout leads to imbalance in hepatic metabolism in F1 generation, the mechanism of which requires further discussion.

## 1 Introduction

In recent years, metabolic syndrome, including obesity, hypertension, dyslipidemia, insulin resistance and other major diseases, has accounted for a large proportion that of threat to human health (Hess et al., 2017). Presently, numerous studies focus on both the human metabolic syndrome, as well as health of the offspring. Notably, early life circumstances can have a wide range of effects on the long-term health of offspring. According to a report, maternal obesity and gestational diabetes mellitus (GDM) are associated with a risk of obesity and diabetes in offspring (Reynolds et al., 2010). Maternal insulin resistance impairs glucose homeostasis and lipid metabolism in male offspring (Isganaitis et al., 2014). These studies revealed that early life environment affects the characteristics of offspring, and their tendency to develop diseases such as metabolic syndrome.

DNA methyltransferase 3B (DNMT3B), a member of the DNA methyltransferase (DNMTs) family, functions as *de novo* methyltransferase. It is able to establish a new methylation pattern in unmethylated DNA and is essential for embryonic development (Meng et al., 2015; Duymich et al., 2016). DNA methylation, a key epigenetic mechanism, is involved in the modulation of gene expression. It is a process catalyzed by DNMTs, during which organisms use s-adenosine methionine (SAM) as a methyl donor to transfer methyl to cytosine residues in DNA cytosine/guanine enrichment area (called CpG island) (Law and Jacobsen, 2010; Jones, 2012).

To an extent, DNA methylation can be used as an evaluation and indication criteria to explain the etiology of metabolic syndrome. It has been reported that differential DNA methylation could explain the link between obesity and insulin metabolism (Liu et al., 2019). A study identified certain DNA methylation regions at birth that are linked to metabolic health like obesity or insulin sensitivity in later childhood (van Dijk et al., 2018). A few systematic reviews concluded that several differentially methylated CpG sites associated with type 2 diabetes and lipid-related diseases could act as reliable biomarkers and future therapeutic targets (Mittelstrass and Waldenberger, 2018; Walaszczyk et al., 2018). Features of DNA methylation are being increasingly identified in tissues that undergo metabolic alteration in obese and diabetic patients including in liver, adipose tissue, skeletal muscle, and pancreas (Cheng et al., 2018).

Abnormalities in the structure or function of the maternal (F0) endometrium may affect the health of the F1 generation. Studies have shown that perinatal exposure of Sprague-Dawley (SD) rats to a mixture of organophosphorus pesticides resulted in endometrial hyperplasia and thickened uterine walls. More remarkably, its F1 generation exhibited delayed physical development, weakened mental development, abnormal levels of progesterone and testosterone and impaired reproductive functions (Yu et al., 2013). It has been reported that maternal ghrelin deficiency resulted in abnormal growth and proliferation of endometrium, which had adverse effects on reproductive capacity of female offspring (Martin et al., 2011). It is well known that exposure to cadmium can affect uterine development in females. In rats exposed to cadmium during pregnancy, endometrial hyperplasia occurred in the female F1 generation and ovarian hormone disorder occurred in the female F2 generation (Li et al., 2018). Male F1 generation showed impaired development and function of leydig cells (Tian et al., 2018). Endometrial inflammatory factors and lipid homeostasis regulators were significantly increased in obese mares, and the uterine environment was changed, which affected the transcriptional expression of genes related to lipid homeostasis, mitochondria and endoplasmic reticulum stress in their embryos, which may have potential long-term effects on the development of offspring (Sessions-Bresnahan et al., 2018). In our previous study, we found that conditional knockout of maternal endometrial *DNMT3B* affected embryo implantation by impairing endometrial decidualization, and the newborn offspring weighed slightly less than the control group (Long et al., 2021). Nevertheless, the effect of maternal endometrial *DNMT3B* deficiency on the health of F1 generation has been rarely reported in literature. Therefore, in this study, we investigated the effects of maternal endometrial *DNMT3B* conditional knockout on the development and metabolism of F1 generation, in an attempt to understand the potential adverse epigenetics effects of *DNMT3B* deletion.

## 2 Materials and methods

### 2.1 Animals and tissue collection

**FIGURE 1:**
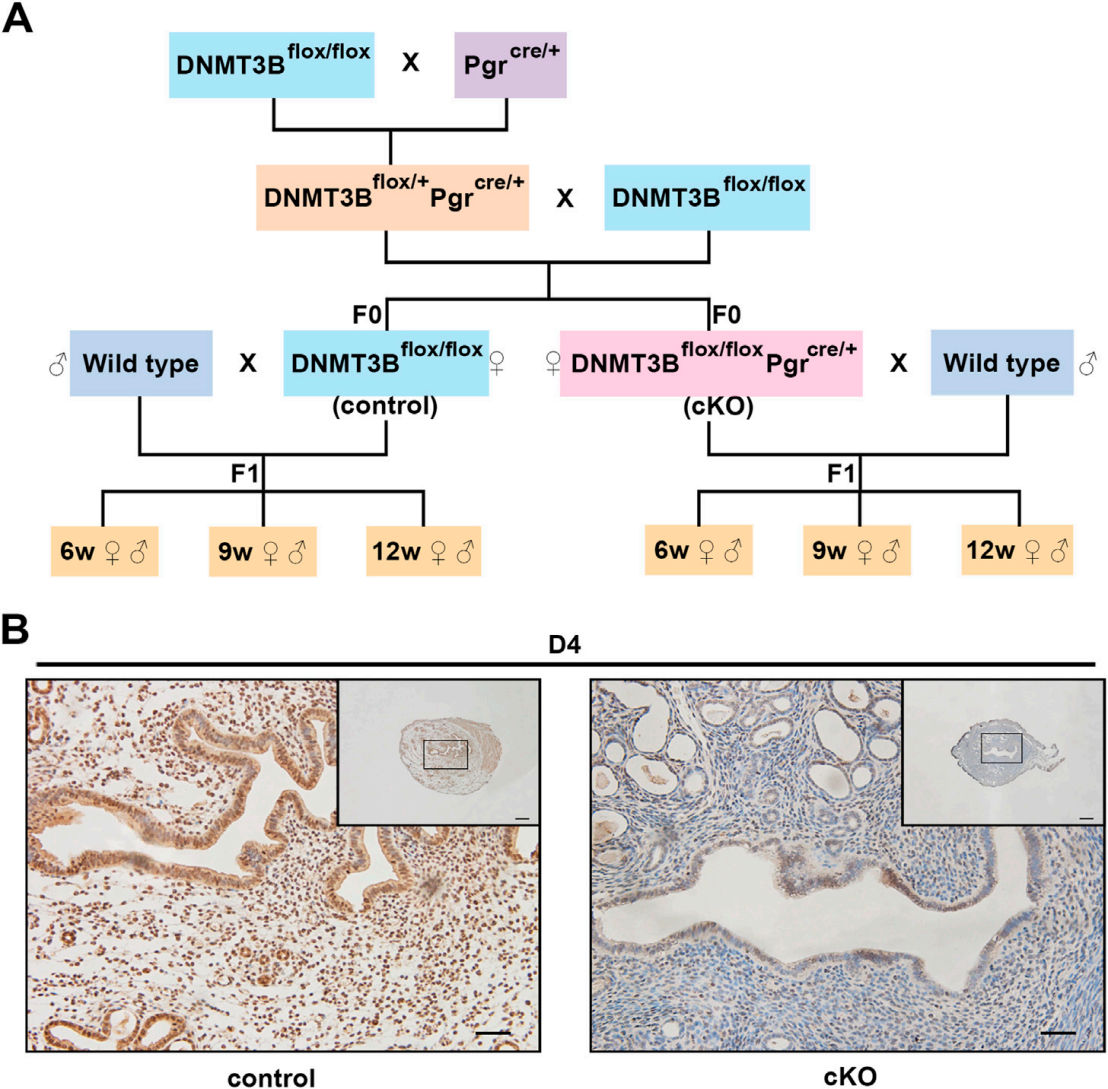
The construction and verification of endometrial *DNMT3B* conditional knockout female mice **(A)** *DNMT3B* ^*flox/flox*^ female mice were mated with *Pgr* ^*Cre/+*^ male mice to acquire *DNMT3B* ^*flox/+*^ *Pgr* ^*Cre/+*^ male mice. Then, *DNMT3B* ^*flox/+*^ *Pgr* ^*Cre/+*^ male mice were mated with *DNMT3B* ^*flox/flox*^ female mice to acquire *DNMT3B* ^*flox/flox*^ (control) and *DNMT3B* ^*flox/flox*^ *Pgr* ^*Cre/+*^ (cKO) female mice. Control or cKO female mice (7–10 weeks old) were mated with normal pubescent C57BL/6 male mice (wild type). Thus, the F1 generation was acquired and divided into three different life stages: 6-week-old (juvenile), 9-week-old (sub-adult) and 12-week-old (adult). **(B)** The expression of uterine endometrial DNMT3B was detected on day 4 using immunohistochemistry. D4: day 4 of pregnancy. Black rectangular box represented magnified area. Scale bar: the larger image is 100μm, and the smaller image is 200 μm.

C57BL/6 *DNMT3B* ^*flox/flox*^ mice were purchased from Riken BioResource Research Center in Japan (RBRC03733). *DNMT3B* ^*flox/flox*^ female mice were mated with progesterone receptor Cre knockin (*Pgr* ^*Cre/+*^) (Soyal et al., 2005) male mice to acquire *DNMT3B* ^*flox/+*^ *Pgr* ^*Cre/+*^ male mice. Then, *DNMT3B* ^*flox/+*^ *Pgr* ^*Cre/+*^ male mice were mated with *DNMT3B* ^*flox/flox*^ female mice to acquire *DNMT3B* ^*flox/flox*^ (control) and *DNMT3B* ^*flox/flox*^ *Pgr* ^*Cre/+*^ (cKO) female mice. Control (n = 4) or cKO (n = 5) female mice (7–10 weeks old) were mated with normal pubescent C57BL/6 male mice to establish pregnancy. Thus, the F1 generation of the aforementioned two mice strains was acquired. The morning of when the presence of the vaginal plug was noted as day 1 of pregnancy (D1). To thoroughly examine the growth and development in F1 generation of control and cKO female mice, we divided the male and female F1 generation into three different life stages: 6-week-old juvenile, 9-week-old sub-adult and 12-week-old adult (Figure 1A). The aforementioned three life stages were assigned to the F1 generation at random. The mice were anesthetized with 0.2 ml of 2% lidocaine by intramuscular injection and liver tissue and serum were collected from each group following decapitation. Liver viscera index was calculated by liver wet weight divided by body weight times 100%. Part of the liver tissue was stored at −80°C, and the rest was fixed in 4% paraformaldehyde. All the animals were fed in germ-free cages at a constant temperature under a 12-h light/12-h dark cycle at the Animal Facility of Chongqing Medical University. Animal experiments were approved by the Ethics Committee of Chongqing Medical University (approval No. 2015–115).

### 2.2 Metabolic level and hormone testing

Blood was drawn from the tail vein at 8:00 a.m. following 12 h of overnight fasting. During fasting, the mice were given free access to water. Fasting blood glucose (FBG) levels were measured and glucose tolerance test (GTT) was performed using a glucometer (Abbott FreeStyle Freedom Lite). Mice were intraperitoneally treated with glucose at dosage of 2 g of glucose/kg body weight after overnight fast. Blood samples were taken from the tail vein before (0 min) and 15, 30, 60, and 120 min after glucose administration. FBG and GTT results were based on continuous detection in the same mice. The area under the curve (AUC) was calculated for glucose clearance following GTT by the trapezoidal method.

Serum lipids such as triglyceride and total cholesterol were detected using blood drawn from the mice fundus venous plexus after being anesthetized. The serum was obtained by refrigerated centrifugation and stored at −80°C. The level of serum triglyceride was measured by glycerol phosphate oxidase-peroxidase-4-aminoantipyrine (GPO-PAP) method using a commercial kit (A110-1-1, Nanjing Jiancheng Bioengineering Institute, Nanjing, China). The level of serum total cholesterol was measured by cholesterol oxidase-peroxidase-4-aminoantipyrine (COD-PAP) method using a commercial kit (A111-1-1, Nanjing Jiancheng Bioengineering Institute, Nanjing, China) according to the manufacturer’s instructions. Very-low-density lipoprotein (VLDL) was calculated using Friedewald’s formula (Friedewald et al., 1972).

### 2.3 Oil red O staining

Fresh liver tissues were fixed in 4% paraformaldehyde for 24 h and then dehydrated in 30% sucrose solution for approximately 12 h. Dehydrated liver tissues were embedded in O.C.T compound (Tissue-Tek, Japan) and stored at −80°C until frozen sectioning. Frozen sections were either stored at −20°C for a while or dried directly for 5 min, rinsed with 60% isopropanol for 5 s, stained with oil red O solution (G1260, Solarbio, Beijing, China) for 60 min, then rinsed with 60% isopropanol for 2–3 s and stained with hematoxylin solution (Solarbio, Beijing, China) for 3 min. Finally, the sections were sealed with gelatin glycerin (Solarbio, Beijing, China) and examined under the microscope (Model: BX43F, Olympus, Tokyo, Japan).

### 2.4.H&E staining

Fresh liver tissues were fixed in 4% paraformaldehyde for 6 h, dehydrated in an alcohol series (75%, 85%, 95%, and 100%), and then embedded in hard wax before sectioning. Following gradient alcohol dehydration, sections were stained with esosin solution (Solarbio, Beijing, China) and hematoxylin dye (Solarbio, Beijing, China). Finally, the sections were sealed using neutral gum and examined under the microscope (Model: BX43F, Olympus, Tokyo, Japan). The results were observed using blinding.

### 2.5 Immunohistochemistry

The procedure for embedding and dehydration of fresh uterus and liver tissues was identical to the one used in H&E staining. Paraffin sections were treated with ethylenediaminetetraacetic acid (EDTA) for antigenic thermal repair. Hydrogen peroxide was used to eliminate endogenous interference. Goat serum (10%, Beyotime Biotechnology, Shanghai, China) was used to block the expression of non-specific antibodies. The paraffin sections were treated with the DAB color reagent kit (SP‐9000, Zhongshan Biosciences Inc., Guangdong, China). The following antibodies were used: DNMT3B rabbit polyclonal antibody (1:100, Abcam) and CPT1A rabbit polyclonal antibody (1:100, Proteintech).

### 2.6 Western blotting

Liver tissues were lysed with ice-cold radio-immunoprecipitation assay (RIPA) buffer (Beyotime Biotechnology, Shanghai, China) containing a protease inhibitor cocktail and phosphatase inhibitor cocktail (Bimake, United States), boiled in 5X sodium dodecyl sulfate (SDS) loading buffer (Beyotime Biotechnology, Shanghai, China) for 10 min and immediately stored at −80°C until further use. Protein concentrations were determined by the bicinchoninic acid assay (BCA) kit (Beyotime Biotechnology, Shanghai, China) according to the manufacturer’s instruction. Equal amounts of protein (20 μg) were subjected to SDS-PAGE and transferred onto polyvinylidene difluoride (PVDF) membranes (1620263, Bio-Rad Laboratories, Shanghai, China). The membranes were blocked with 5% skimmed milk for 90 min at 37°C and incubated with appropriate antibodies overnight at 4°C. The following antibodies were used: CPT1A rabbit polyclonal antibody (1:1,000; Proteintech) and PPARα rabbit polyclonal antibody (1:1,000; Proteintech). β-actin (1:1,000; Boster) was used as an internal reference. Bands obtained through western blotting were detected by chemiluminescent reaction using Immobilon ECL Ultra Western HRP Substrate (WBULS0500, Millipore). Image collection and densitometry analysis were quantified using Quantity One version 4.5.0 analysis software (Bio-Rad). Densities were normalized to the control and relative folds were normalized to β-actin.

### 2.7 Determination of liver lipid and lipid peroxidation levels

Fresh liver tissues were collected and stored at −80°C. Liver triglyceride concentration was measured by glycerol phosphate oxidase-peroxidase-4-aminoantipyrine (GPO-PAP) method using a commercial kit (E1013, Applygen Technologies Inc., Beijing, China) and malondialdehyde (MDA) concentration was measured by thibabituric acid (TBA) method using corresponding assay kit (A003-1, Nanjing Jiancheng Bioengineering Institute, Nanjing, China) according to the manufacturer’s instructions. Each tissue sample was added to a 96-well plate, and the absorbance of each well was measured at the specified working wavelength. Based on the standard absorbance and standard curve and the protein concentration of each tissue sample, the triglyceride concentration and MDA concentration of each sample was calculated for subsequent statistical analysis.

### 2.8 Statistics

All experiments were independently repeated at least thrice. Data was expressed as mean ± SEMs. Data with normal distribution were analyzed by two-tailed Student’s t-test. Non-parametric test was used for data that did not follow normal distribution. Two-way ANOVA was used to evaluate GTT and sex-dependent effects. SPSS statistics 17.0 was used for data statistical analysis, and GraphPad Prism 7.0 was used for making statistical charts. Values were considered significant at *p* < 0.05.

## 3 Results

### 3.1 Glucose homeostasis was impaired in the F1 generation of endometrial *DNMT3B* conditional knockout female mice

**FIGURE 2:**
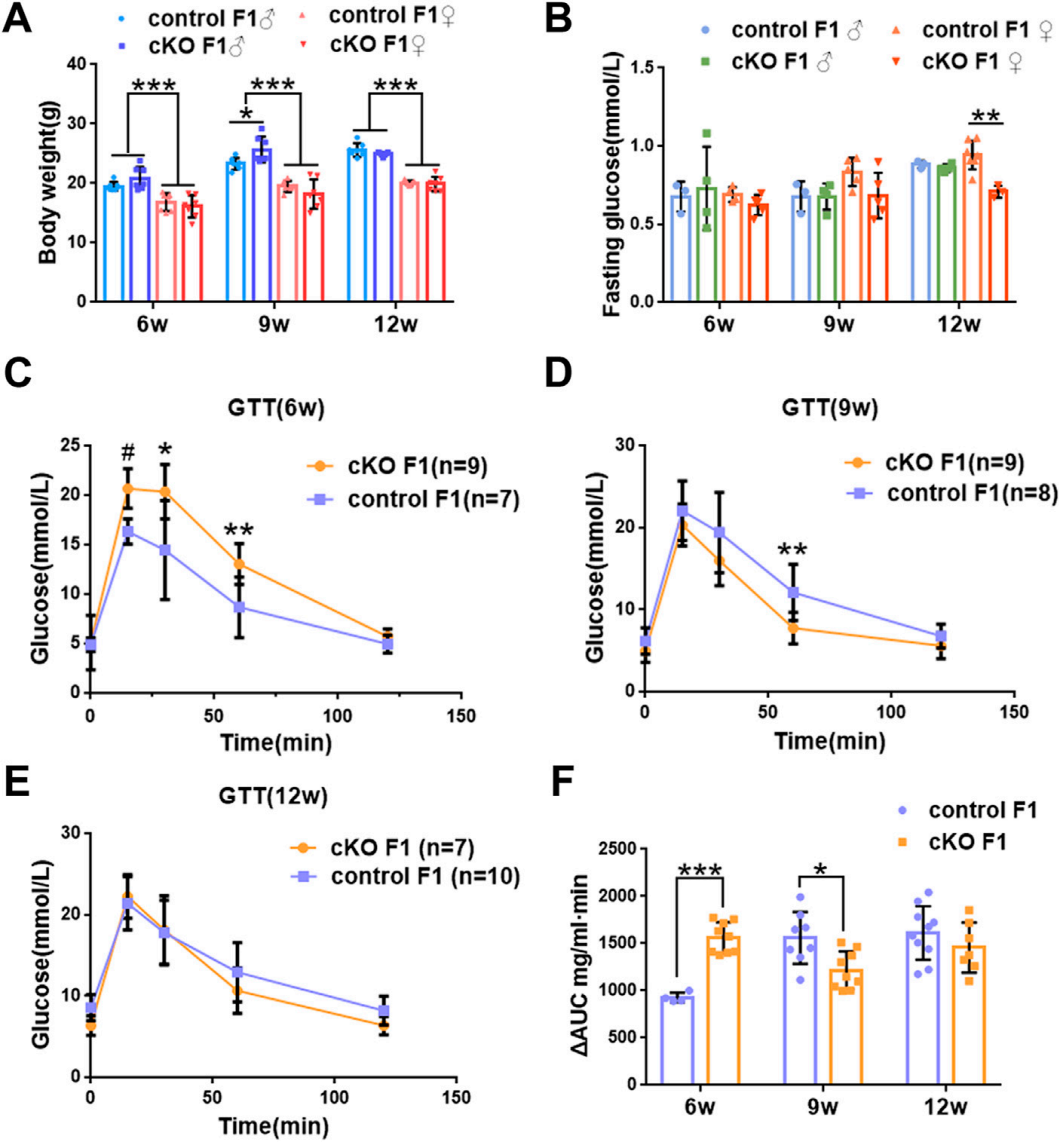
Glucose homeostasis was impaired in the F1 generation of endometrial *DNMT3B* conditional knockout female mice **(A)** The body weight of the F1 generation was recorded continuously at 6-weeks, 9-weeks and 12-weeks of age (n = 7). **(B)** Fasting blood glucose level of the F1 generation at 6-weeks, 9-weeks and 12-weeks of age (n = 7–9). **(C–E)** Glucose tolerance in the F1 generation at 6-weeks, 9-weeks and 12-weeks of age was measured continuously (n = 7–10). **(F)** The area under the curve of glucose tolerance in control and cKO F1 mice at 6-weeks, 9-weeks and 12-weeks of age. Results are presented as mean ± SEM. *p < 0.05, **p < 0.01, *** and #*p* < 0.001. GTT, glucose tolerance test. AUC, the area under the curve.

**FIGURE 3:**
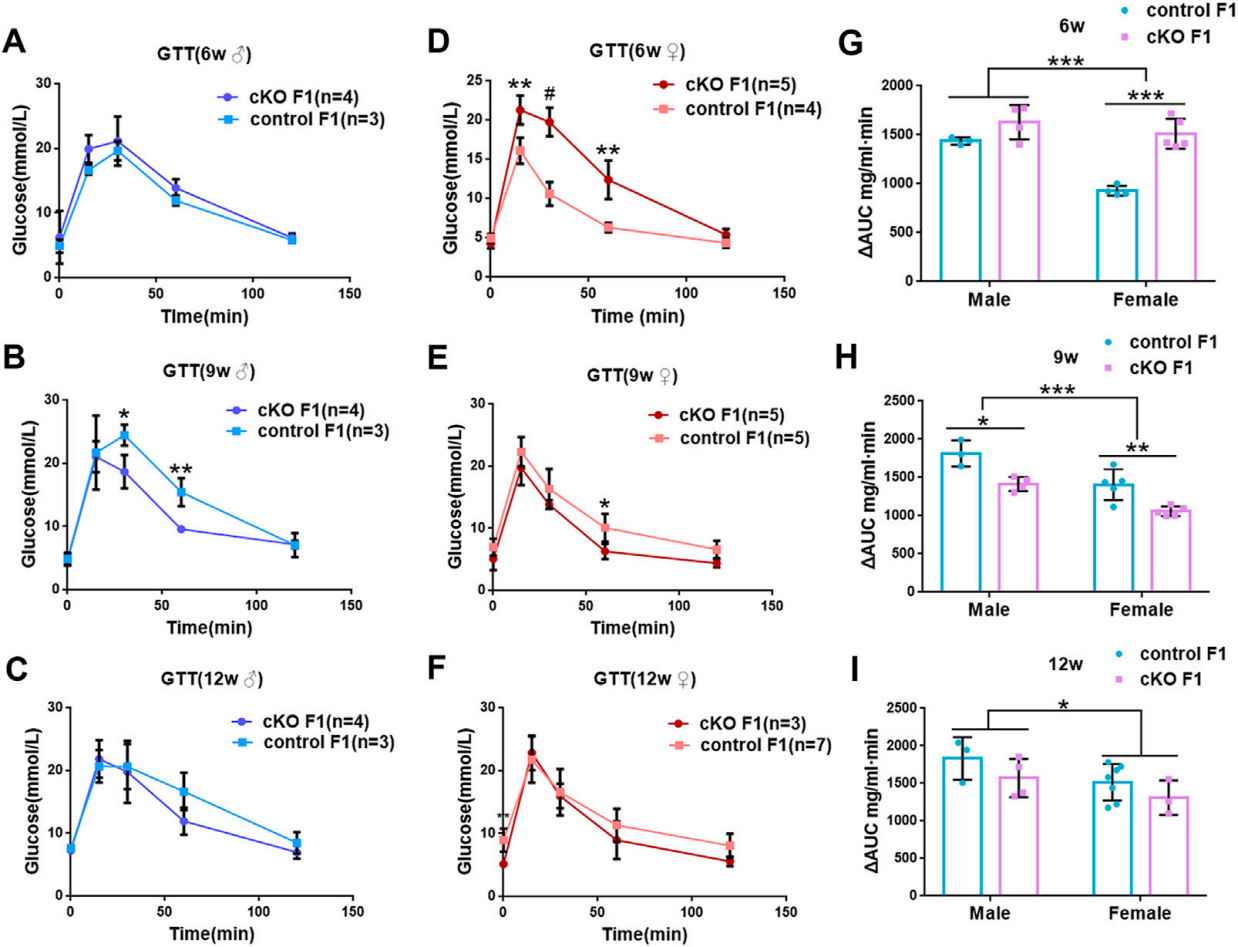
Glucose tolerance was measured by GTT in the male and female F1 generation of control and cKO female mice **(A–C)** Glucose tolerance in control and cKO F1 male mice was measured at 6-weeks, 9-weeks and 12-weeks of age (n = 3–4). **(D–F)** Glucose tolerance in control and cKO F1 female mice was measured at 6-weeks, 9-weeks and 12-weeks of age (n = 3–7). **(G–I)** The area under the curve of glucose tolerance in control and cKO F1 male and female mice at 6-weeks, 9-weeks and 12-weeks of age. Results are presented as mean ± SEM. *p < 0.05, **p < 0.01, *** and #*p* < 0.001. GTT, glucose tolerance test. AUC, the area under the curve.

We first acquired endometrial *DNMT3B* conditional knockout female mice (cKO) and their control counterparts (control), and their F1 offspring, as described in the methods (Figure 1A). To validate if *DNMT3B* was knocked out in the uterus of the aforementioned female mice, the expression of uterine DNMT3B was detected. The result indicated that DNMT3B was successfully knocked out in the cKO female mice on D4 (Figure 1B). To understand the development and metabolism of the F1 generation, the body weight was recorded continuously at the ages of 6-weeks, 9-weeks and 12-weeks. We found that cKO F1 male mice at 9-weeks of age had significantly increased body weight compared to control male F1 mice (*p* < 0.05), and significant effects of sex were observed at different weeks of age (6w: *F*_1,24_ = 38.46, *p* < 0.001; 9w: *F*_1,24_ = 71.19, *p* < 0.001; 12w: *F*_1,24_ = 257.9, *p* < 0.001) (Figure 2A). The FBG levels of F1 generation were measured and results showed that at 12-weeks of age, FBG levels of cKO female F1 mice was significantly decreased compared to control female F1 mice (*p* < 0.01). However, no significant difference was found in both sexes (6w: *F*_1,12_ = 0.3924, *p* = 0.5427; 9w: *F*_1,13_ = 2.276, *p* = 0.1553; 12w: *F*_1,13_ = 1.75, *p* = 0.2087) (Figure 2B). The GTT results showed glucose tolerance of cKO F1 mice differed from the control F1 mice. At 6-weeks of age, blood glucose level of cKO F1 mice was significantly elevated following glucose injection, and the AUC was significantly higher than that of the control F1 mice (*p* < 0.001). But at 9-weeks of age, blood glucose level of cKO F1 mice was significantly reduced at 60 min compared to control F1 mice, and the AUC was significantly lower than that of the control F1 mice (*p* < 0.05). At 12-weeks of age, blood glucose level of these two groups was similar (Figures 2C–F). This indicated that glucose clearance was significantly delayed in cKO F1 mice at 6-weeks of age, but was restored gradually between 9-weeks and 12-weeks of age. Similar results were found in male and female F1 mice (Figures 3A–F). Notably, at 6-weeks of age, cKO female F1 mice exhibited the most impaired glucose tolerance (*p* < 0.001) (Figures 3D,G). Sex-dependent effect analysis showed that there were significant effects of sex on the glucose tolerance at different weeks of age (6w: *F*_1,12_ = 23.36, *p* < 0.001; 9w: *F*_1,13_ = 29.51, *p* < 0.001; 12w: *F*_1,13_ = 5.066, *p* < 0.05) (Figures 3G–I). Overall, glucose homeostasis was impaired in the F1 generation of cKO female mice compared to control female mice.

### 3.2 Liver function was impaired in the F1 generation of endometrial *DNMT3B* conditional knockout female mice

**FIGURE 4:**
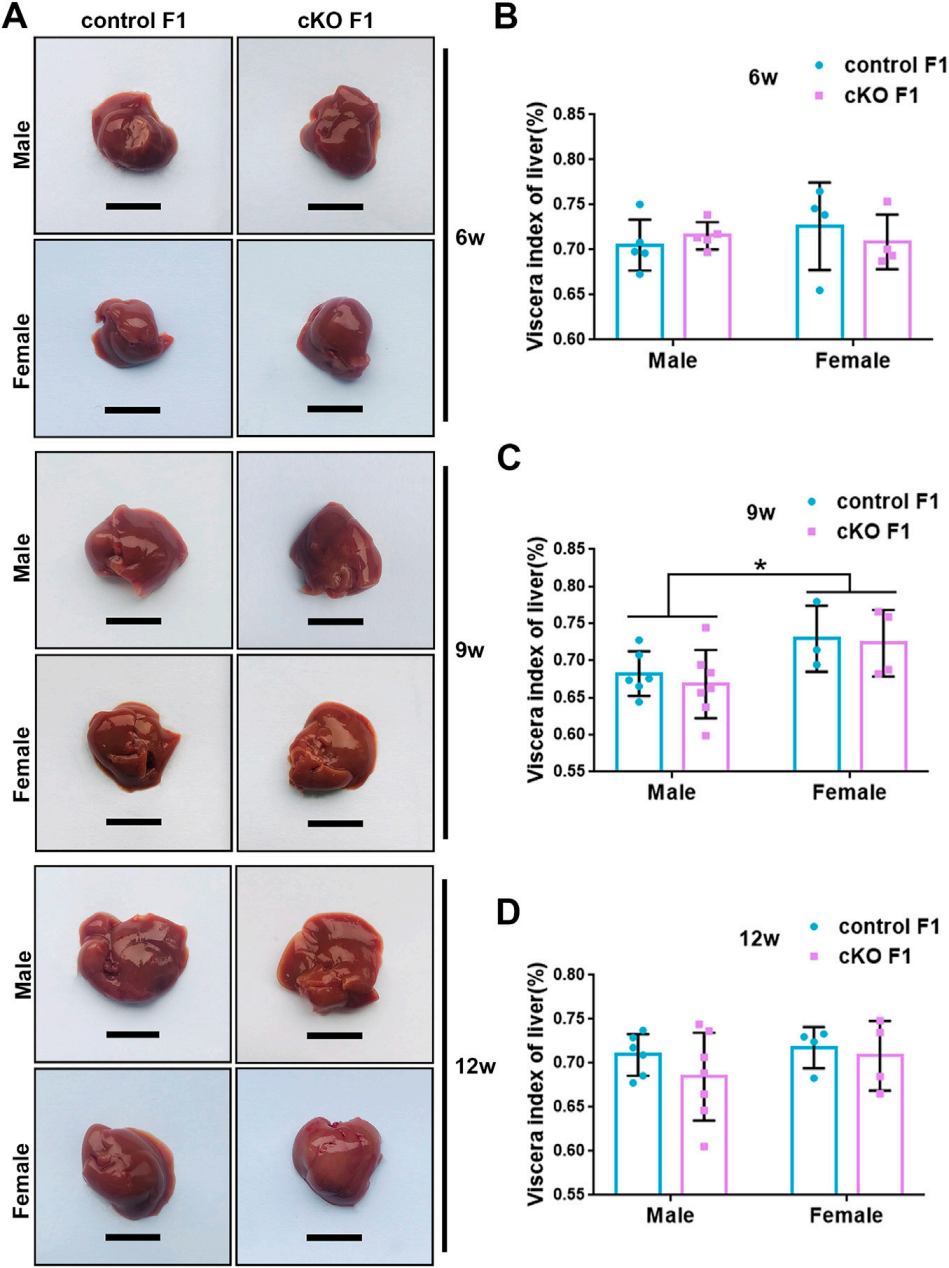
Liver morphology of the F1 generation of endometrial *DNMT3B* conditional knockout female mice **(A)** Liver morphology of control and cKO F1 male and female mice at 6-weeks, 9-weeks and 12-weeks of age. **(B–D)** Viscera index of liver was calculated in control and cKO F1 male and female mice at 6-weeks, 9-weeks and 12-weeks of age (n = 9–11). Results are presented as mean ± SEM. Scale bar: 1.5 cm *p < 0.05.

**FIGURE 5:**
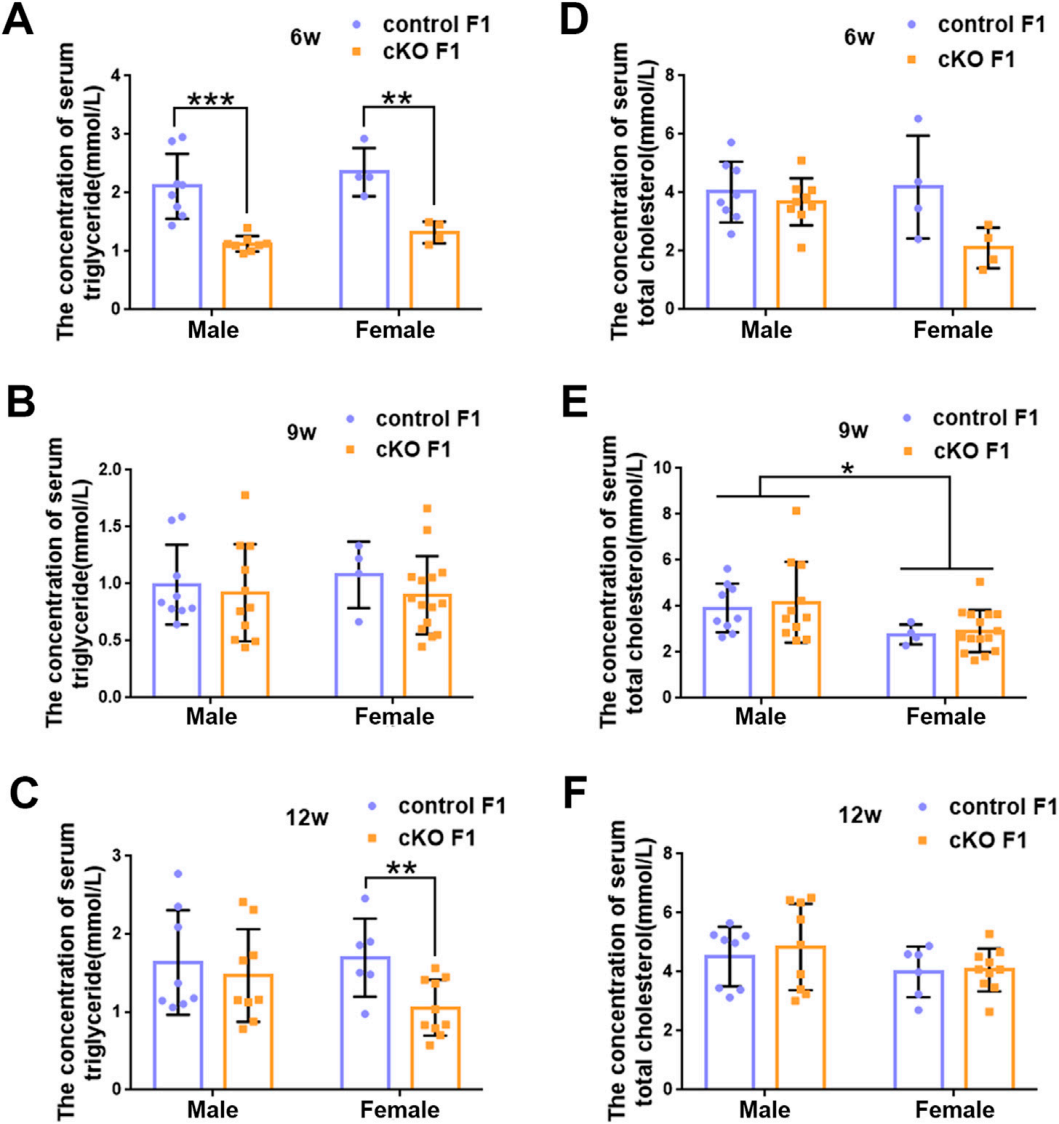
The concentration of serum lipid was measured in F1 generation of endometrial *DNMT3B* conditional knockout female mice **(A–C)** Serum triglyceride levels were measured in control and cKO F1 male (n = 8–11) and female (n = 4–15) mice at 6-weeks, 9-weeks and 12-weeks of age. **(D–F)** Serum total cholesterol levels were measured in control and cKO F1 male (n = 8–11) and female (n = 4–15) mice at 6-weeks, 9-weeks and 12-weeks of age. Results are presented as mean ± SEM. *p < 0.05, **p < 0.01, ***p < 0.001.

**FIGURE 6:**
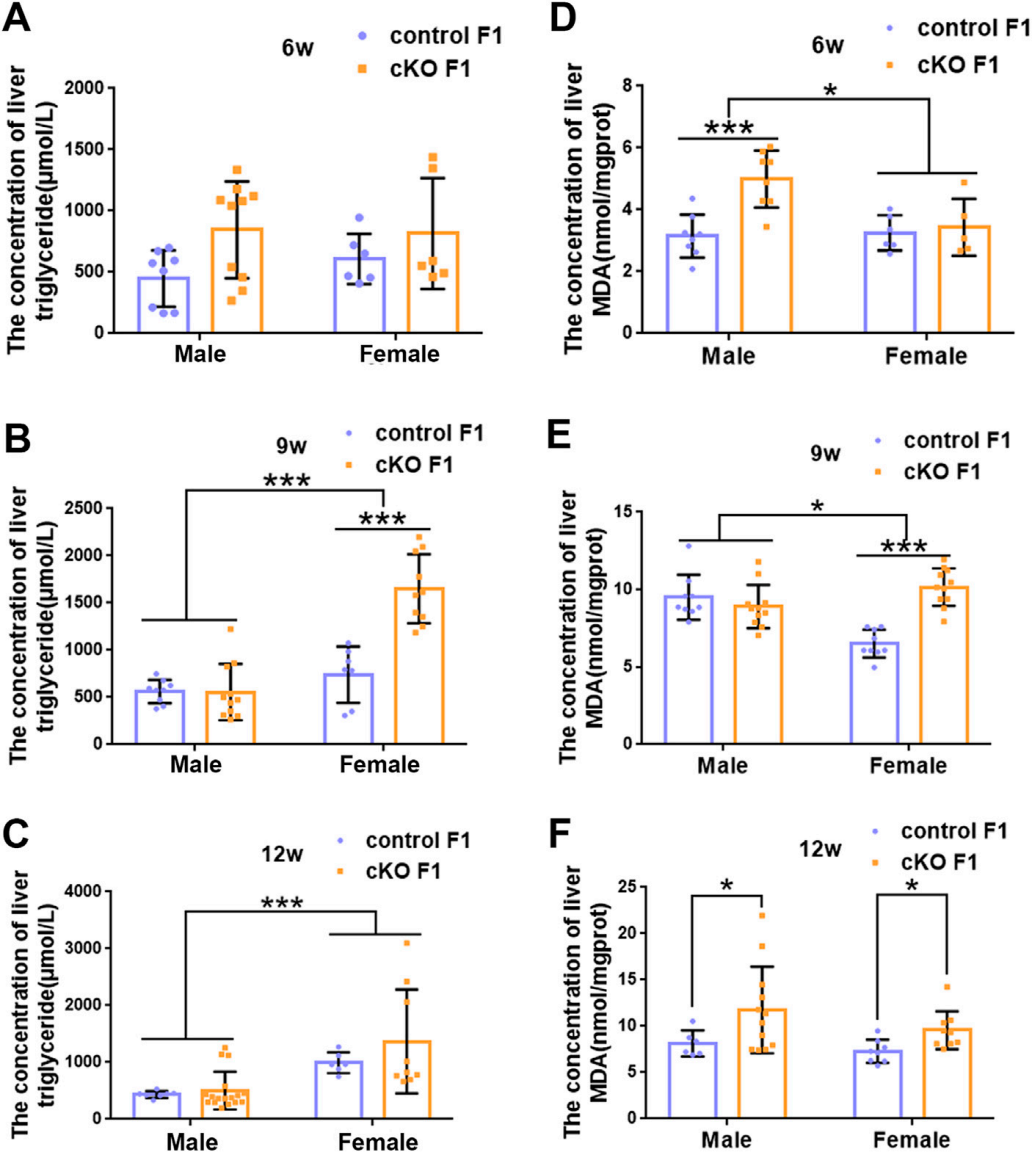
The concentration of liver lipid was measured in F1 generation of endometrial *DNMT3B* conditional knockout female mice **(A–C)** The concentration of liver triglyceride was measured in control and cKO F1 male (n = 8–17) and female (n = 6–10) mice at 6-weeks, 9-weeks and 12-weeks of age. **(D–F)** The concentration of liver malondialdehyde was measured in control and cKO F1 male (n = 8–12) and female (n = 5–11) mice at 6-weeks, 9-weeks and 12-weeks of age. Results are presented as mean ± SEM. MDA, malondialdehyde. *p < 0.05, ***p < 0.001.

Liver acts as a major glucose regulator in both humans and mice. Hepatocytes uptake and release blood glucose through the plasma membrane transporter, glucose transporter 2 (GLUT2), which regulates the body’s blood-glucose balance (Rui, 2014). Moreover, the liver plays a crucial role in the regulation of lipid metabolism. Long-chain fatty acids are converted into triacylglycerol, phospholipids, and cholesterol esters in hepatocytes, and are then stored in lipid droplets and membrane structures, or secreted into circulation as VLDL particles (Rui, 2014). Considering that glucose metabolism was disrupted in cKO F1 mice, we then examined liver function in the F1 generation. At 6-weeks, 9-weeks or 12-weeks of age, the morphology of liver revealed no significant difference between control and cKO F1 male or female mice (Figure 4A). Liver viscera index was calculated and showed no difference in either male or female F1 generation of control and cKO mice at different weeks of age (Figures 4B–D). There was a significant sex-dependent effect on liver viscera index at 9-weeks of age (*F*_1,16_ = 6.89, *p* < 0.05) (Figure 4C). Moreover, compared to control F1 mice, serum triglyceride levels significantly reduced in cKO male (*p* < 0.001) and female (*p* < 0.01) F1 mice at 6-weeks of age and cKO female (*p* < 0.01) F1 mice at 12-weeks of age, and a decreasing trend was observed in 9-week-old cKO F1 male (*p* = 0.518) and female (*p* = 0.354) mice and 12-week-old cKO F1 male (*p* = 0.630) mice (Figures 5A–C, Tables 1–3). Similar results were found in the concentration of serum VLDL (Tables 1–3). There was no significant difference in serum total cholesterol level in F1 mice at 6-weeks, 9-weeks and 12-weeks of age (Figures 5D–F; Tables 1–3). However, there was a significant sex-dependent effect at 9-weeks of age (*F*_1,35_ = 7.386, *p* < 0.05) (Figure 5E). The concentration of liver triglyceride significantly increased in cKO female F1 mice at 9-weeks of age (*p* < 0.001) (Figure 6B). Although, no significant difference was noted in other groups, cKO F1 groups had a few individual mice with elevated liver triglyceride concentrations compared to control F1 mice (Figures 6A–C). At 9-weeks and 12-weeks of age, there was a significant effect of sex on liver triglyceride concentrations (9w: *F*_1,33_ = 43.83, *p* < 0.001; 12w: *F*_1,35_ = 17.2, *p* < 0.001) (Figures 6B,C). Additionally, the concentration of liver MDA was measured and found a significant increase in 6-week-old cKO F1 male mice (*p* < 0.001) as well as 9-week-old cKO F1 female mice (*p* < 0.001) and 12-week-old cKO F1 male (*p* < 0.05) and female (*p* < 0.05) mice. A significant effect of sex on Liver MDA was observed at 6-weeks and 9-weeks of age (6w: *F*_1,23_ = 5.481, *p* < 0.05; 9w: *F*_1,36_ = 4.616, *p* < 0.05) (Figures 6D–F). Overall, these findings suggested that in cKO F1 mice, the ability of the liver to metabolize maybe impaired, and triglyceride metabolism was abnormal, posing a risk of triglyceride accumulation in the liver, which might lead to the development of fatty liver. Elevated MDA level could destroy biofilms, thereby impairing the metabolic functions of the liver.

**TABLE 1:** The serum lipid concentrations of control and cKO F1 mice at 6-weeks of age.

	Male		Female	
	control F1	cKO F1	control F1	cKO F1
Triglyceride (mmol/L)	2.112 ± 0.196	1.129 ± 0.047^b^	2.355 ± 0.306	1.323 ± 0.093^a^
VLDL (mmol/L)	0.422 ± 0.039	0.226 ± 0.009^b^	0.471 ± 0.041	0.265 ± 0.019^a^
Total cholesterol (mmol/L)	4.023 ± 0.367	3.716 ± 0.301	4.196 ± 0.878	2.107 ± 0.350

Results are expressed as mean ± SEM.

^a^ *p* < 0.01.

^b^ *p* < 0.001.

**TABLE 2:** The serum lipid concentrations of control and cKO F1 mice at 9-weeks of age.

	Male		Female	
	control F1	cKO F1	control F1	cKO F1
Triglyceride (mmol/L)	0.992 ± 0.117	0.922 ± 0.129	1.079 ± 0.146	0.900 ± 0.088
VLDL (mmol/L)	0.198 ± 0.023	0.184 ± 0.026	0.216 ± 0.029	0.180 ± 0.018
Total cholesterol (mmol/L)	3.919 ± 0.353	4.171 ± 0.530	2.773 ± 0.216	2.924 ± 0.238

Results are expressed as mean ± SEM.

**TABLE 3:** The serum lipid concentrations of control and cKO F1 mice at 12-weeks of age.

	Male		Female	
	control F1	cKO F1	control F1	cKO F1
Triglyceride (mmol/L)	1.635 ± 0.237	1.469 ± 0.198	1.698 ± 0.204	1.057 ± 0.113^a^
VLDL (mmol/L)	0.327 ± 0.047	0.294 ± 0.040	0.340 ± 0.041	0.211 ± 0.023^a^
Total cholesterol (mmol/L)	4.523 ± 0.358	4.848 ± 0.489	4.003 ± 0.352	4.069 ± 0.229

Results are expressed as mean ± SEM.

^a^ *p* < 0.01.

### 3.3 Compromised liver micromorphology and enhanced lipid droplet deposition were observed in F1 generation of endometrial *DNMT3B* conditional knockout female mice

**FIGURE 7:**
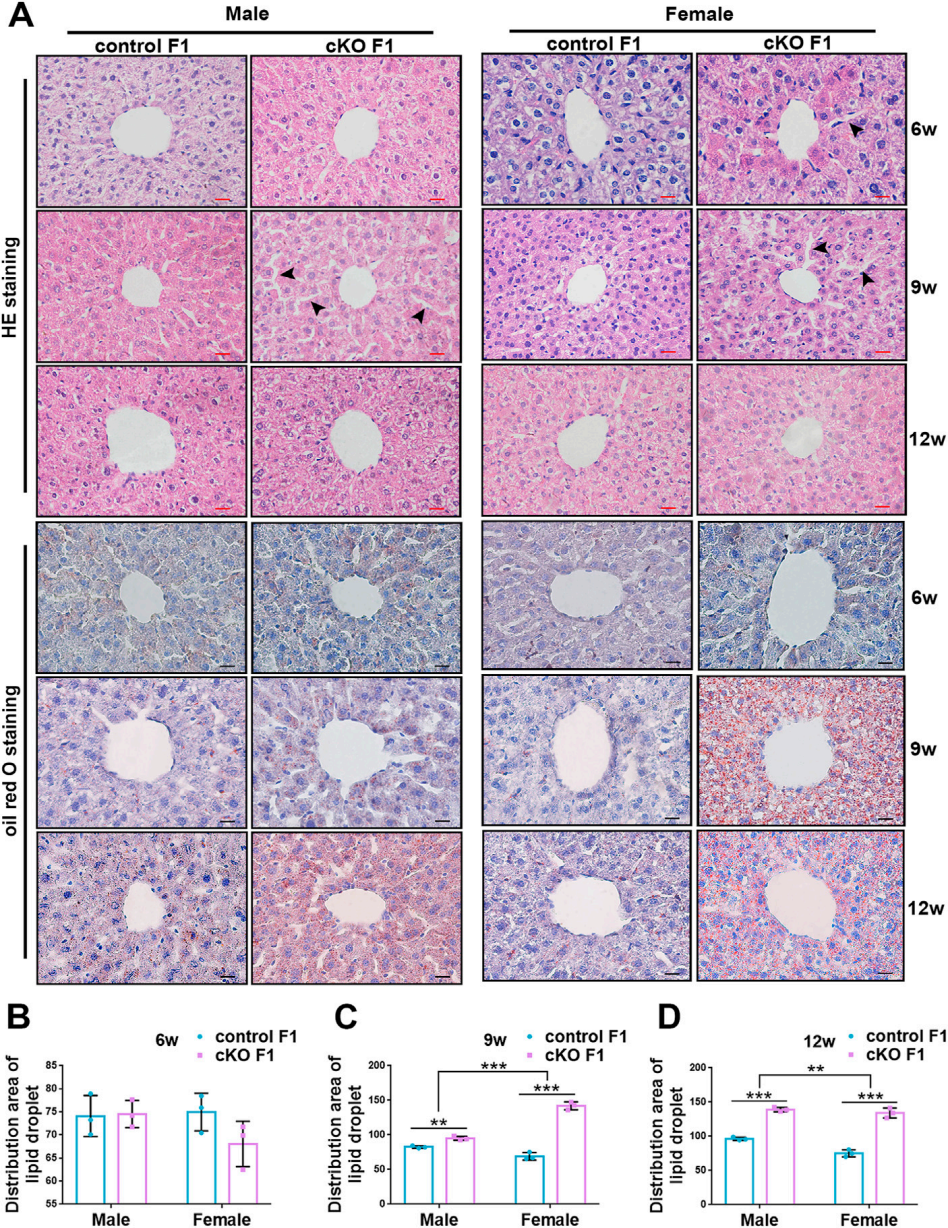
Compromised liver structure and enhanced lipid droplet deposition were observed in F1 generation of endometrial *DNMT3B* conditional knockout female mice. **(A)** Upper three lines: the micromorphology of liver in control and cKO F1 mice at 6-weeks, 9-weeks and 12-weeks of age was detected using H&E staining. Lower three lines: lipid droplets around the central vein of the liver in control and cKO F1 mice at 6-weeks, 9-weeks and 12-weeks of age were detected using oil red O staining. **(B–D)** The statistical results of lipid droplets distribution area (n = 3). Results are presented as mean ± SEM. **p < 0.01, ***p < 0.001. Black arrow: expansion of hepatic cord space. Scale bar: red bar and black bar are 20 μm.

Liver glucose and lipid metabolism is mainly carried out in hepatocytes. The structural integrity of liver and normal morphology and function of hepatocytes form the basis for normal regulation of liver metabolism. To further explore the metabolic function of the liver, we observed liver micromorphology and lipid droplet deposition of control and cKO F1 mice. H&E staining revealed that in cKO F1 female mice at 6-weeks of age and cKO F1 male and female mice at 9-weeks of age, the hepatocytes were loosely arranged and hepatic cords space was enlarged compared to control F1 mice (Figure 7A). Oil red O staining indicated that lipid droplets were abundant and widely distributed around the central vein in cKO F1 mice at 9-weeks and 12-weeks of age, and the effect of sex was significant (9w: *F*_1,8_ = 46.99, *p* < 0.001; 12w: *F*_1,8_ = 21.42, *p* < 0.01) (Figures 7A–D). Overall, liver micromorphology of cKO F1 mice was altered compared to control F1 mice, and lipid droplet deposition was elevated in cKO F1 mice.

### 3.4 The expression of key lipid metabolism factors in the liver was down-regulated in the F1 generation of endometrial *DNMT3B* conditional knockout female mice

**FIGURE 8:**
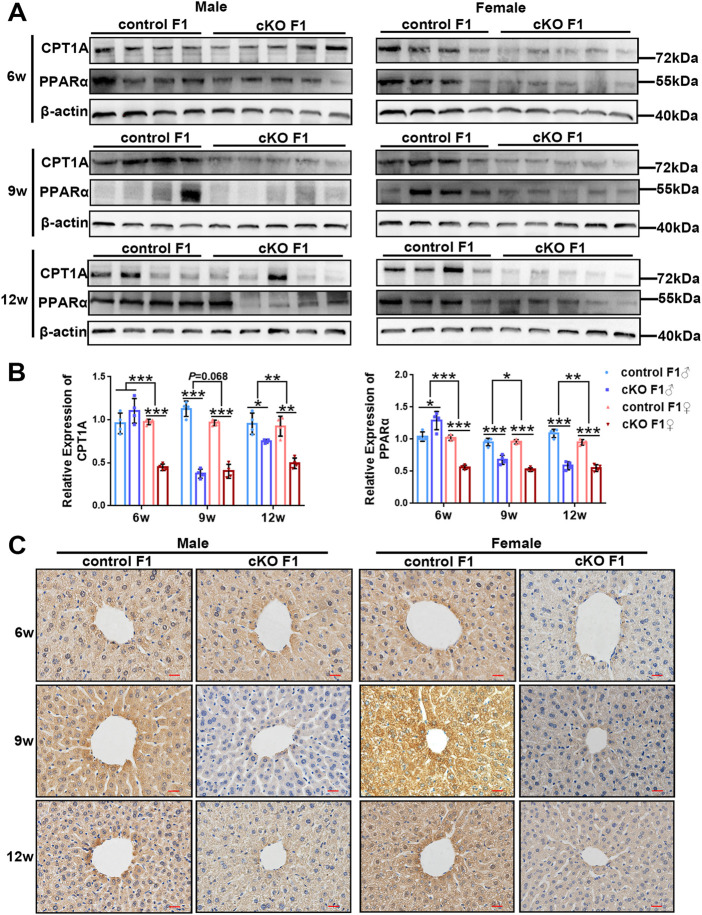
The expression of key lipid metabolism factors in the liver was down-regulated in the F1 generation of endometrial *DNMT3B* conditional knockout female mice **(A)** The expression of CPT1A and PPARα in the liver of control and cKO F1 mice was measured using western blotting at 6-weeks, 9-weeks and 12-weeks of age (n = 4–5). **(B)** Relative expression of CPT1A and PPARα. β-actin was used as the standard to calibrate the gray value of each sample. **(C)** The expression of CPT1A was measured using immunohistochemistry. Results are presented as mean ± SEM. *p < 0.05, **p < 0.01, ***p < 0.001. Scale bar: 20 μm.

To investigate liver lipid metabolism in control and cKO F1 mice, we examined the expression of key lipid metabolism factors in the liver. Carnitine palmitoyl transferase 1A (CPT1A) catalyzes the transfer of the acyl group of the long-chain fatty acid-CoA conjugates to carnitine, an essential step in the mitochondrial uptake of long-chain fatty acids and their subsequent β-oxidation in the mitochondria, which plays an important role in triglyceride metabolism (Prip-Buus et al., 2001; Gobin et al., 2003). Peroxisome proliferator activated receptor alpha (PPARα) regulates the peroxisomal β-oxidation pathway of fatty acids (Laurent et al., 2013). Western blotting showed that CPT1A and PPARα expressions were significantly down-regulated in cKO F1 mice compared to control F1 mice, with the exception of cKO F1 male mice at 6-weeks of age (Figures 8A,B). Results from the two-way between-groups ANOVA revealed a significant effect of sex on CPT1A (6w: *F*_1,12_ = 43.85, *p* < 0.001; 12w: *F*_1,12_ = 9.945, *p* < 0.01) and PPARα (6w: *F*_1,12_ = 79.46, *p* < 0.001; 9w: *F*_1,12_ = 8.324, *p* < 0.05; 12w: *F*_1,12_ = 9.84, *p* < 0.01) expressions at different weeks of age, except the expression of CPT1A at 9-weeks of age (*F*_1,12_ = 4.023, *p* = 0.068) which has a margin of statistical significance (Figure 8B). Immunohistochemistry analysis of CPT1A expression revealed similar results (Figure 8C). Overall, the ability of the liver to metabolize lipids was impaired in cKO F1 mice compared to control F1 mice, indicating that maternal endometrium conditional knockout *DNMT3B* might affect lipid metabolism in F1 generation.

## 4 Discussion

DNA methylation, an epigenetic modification associated with transcriptional silencing, is essential for mammalian development. *De novo* DNA methyltransferases DNMT3A and DNMT3B in conjunction with DNMT3L establish DNA methylation (Schubeler, 2015). DNMT3A and DNMT3B are the main catalytic enzymes that re-establish DNA methylation during embryonic development (Chen and Zhang, 2020). It has been reported that the role of DNA methylation in endometrial biology is directly related to the expression of numerous implantation-related genes (Guo, 2009a; Koukoura et al., 2016). Dyson et al. reported significant difference in methylation patterns of several genes and transcriptional regulators, associated with the pathology of endometriosis and decidualization (Dyson et al., 2014). Additionally, aberrant establishment of methylation patterns have been linked to several endometrial abnormalities such as implantation failure, deviant endometrial pathologies, adenomyosis, and endometriosis (Guo, 2009a; Guo, 2009b; Tao and Freudenheim, 2010). Similarly, in our previous study, we found that maternal endometrial *DNMT3B* conditional knockout affects embryo implantation by impairing endometrial decidualization (Long et al., 2021). Furthermore, we found that conditional knockout of *DNMT3A* in the uterus did not significantly affect endometrial function during embryo implantation (Li et al., 2020). These results suggested that DNMT3B was more essential in the endometrium. Genomic studies revealed that DNMT3B preferentially binds to regions with higher CpG density and is accompanied by co-transcriptional deposition of H3K36me3, thereby playing a role in *de novo* methylation (Baubec et al., 2015).

It is now widely accepted that preconception environmental exposures have a significant impact on offspring health and development *via* epigenetics (Lane et al., 2014). This viewpoint is consistent with the developmental origins of health and disease (DOHAD) hypothesis (Oestreich and Moley, 2017). Additionally, studies have shown that DNA methylation is closely associated with lipid metabolism and adipogenesis, and might explain the relationship between DNA methylation and metabolic disorders (Castellano-Castillo et al., 2019). Liver is a major organ in fatty acid β-oxidation and synthesis of triglyceride during lipid metabolism. Cholesterol and fatty acids utilized in the synthesis of triglycerides, are essential components of animal cell membranes. Moreover, liver also plays a vital role in glucose metabolism. Blood glucose is accessible to hepatocytes *via* glucose transporters, and ATP is produced to provide energy to the body through glycolysis, tricarboxylic acid cycle, and other pathways (Han et al., 2016). Dysregulation of lipid and glucose metabolism causes a variety of disorders such as obesity in humans (Cheng et al., 2019). In our previous work, we reported that maternal endometrial *DNMT3B* conditional knockout exhibited impaired fertility associated with defective endometrial decidualization. The number of embryo implantation sites and the expression of decidualization markers such as homeobox A10 (HOXA10), bone morphogenetic protein 2 (BMP2), matrix metalloproteinase-2 (MMP2), and MMP9 in the endometrium were significantly reduced in cKO F0 female mice (Long et al., 2021). Transcriptome sequencing revealed that the expression of pyruvate kinase gene in the endometrium of cKO F0 female mice was significantly down-regulated (results not shown). PKM2, a pyruvate kinase isoform, is a key protein in glucose and lipid metabolism (Liu et al., 2017). We also observed that the birth weight of cKO F1 mice was slightly lower than that of the control group, which encouraged us to explore the growth and metabolism of the F1 generation (Long et al., 2021).

In this study, we investigated the glucose and lipid metabolism at different ages of male and female F1 generation, which included the offspring of cKO and its control female mice. We focused on three life stages 6-weeks (juvenile), 9-weeks (sub-adult), and 12-weeks (adult) (Nkomozepi et al., 2019). *De novo* methylation gene *DNMT3B* was conditionally knocked out from the endometrial stromal cells of maternal mice, which might cause changes in uterine environment and influence health status of the next generation. During our research, we found the FBG of cKO F1 mice decreased significantly at 12-weeks of age, especially in cKO F1 female mice. Corresponding to this result, GTT of the cKO F1 mice returned to approximately same level as that of the control F1 mice at 12-weeks of age compared to 6-weeks and 9-weeks. Although, the FBG levels of cKO F1 mice was not significantly different from that of control F1 mice at 6-weeks of age, the GTT curve of 6-week-old cKO F1 mice was significantly elevated at 15, 30, and 60 min, indicating impaired blood glucose regulation in cKO F1 mice. At 9-weeks of age, the GTT curve of cKO F1 mice decreased significantly at 30 and 60 min, and finally reached similar level as the control group at 12-weeks of age, suggesting that the blood glucose regulation ability of mice showed gradual recovery. The decrease in GTT curve at 9-weeks of age might be a compensatory regulatory effect, but it did not affect the FBG level of cKO F1 mice. These results suggested that cKO F1 mice were at a greater risk of developing metabolic disorders such as diabetes than control F1 mice. However, body weight only differed in male F1 mice at 9-weeks of age and there was no significant difference in liver viscera index between control and cKO F1 mice. These results suggested that glucose metabolism disorders persisted in cKO F1 mice, even in the absence of obesity or hyperglycemia.

**FIGURE 9:**
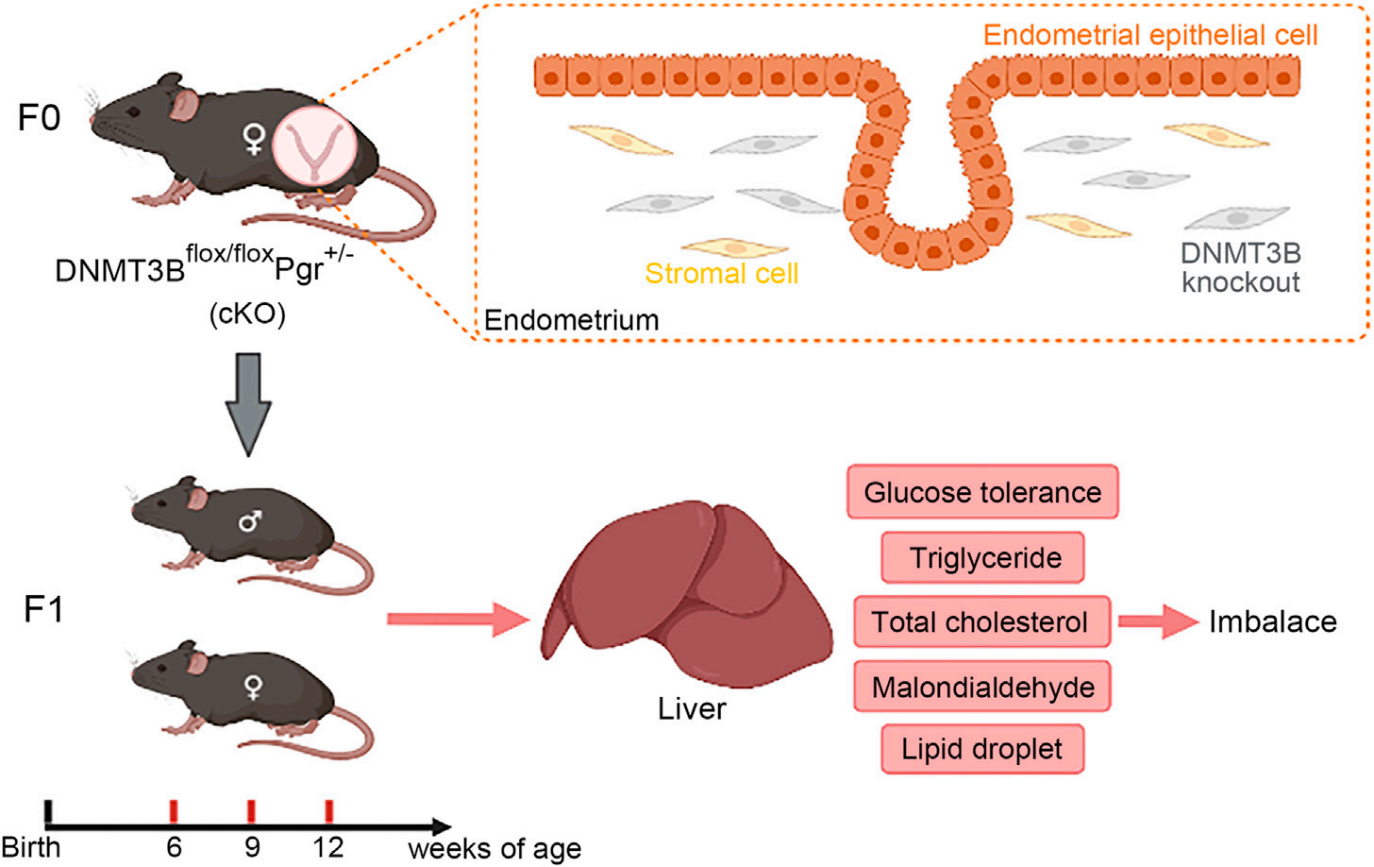
In this study, *de novo* methylation gene *DNMT3B* was conditionally knocked out from the endometrial stromal cells of maternal mice, which might cause changes in uterine environment and influence health status of the next generation. Our results indicated that hepatic glucose and lipid metabolism was abnormal in cKO F1 mice, mainly manifested as impaired glucose tolerance, abnormal serum and liver lipid levels, and increased lipid droplets deposition in the liver, suggesting that cKO F1 mice were at a greater risk of metabolic syndrome than control F1 mice.

Liver is an important organ that regulate glucose and lipid metabolism. Initially, we observed the micromorphology of liver to examine the presence of alterations between control and cKO F1 mice. Compared to the control F1 mice, the liver structure of cKO F1 mice was relatively loose, and the hepatic cord space showed expansion. Changes in liver structure might have an effect on liver functions in cKO F1 mice. We then examined lipid metabolism in the liver and found an increase in lipid droplet deposition around the central hepatic vein in cKO F1 mice, especially at 9-weeks and 12-weeks of age. Both CPT1A and PPARα are the key factors of β-oxidation in lipid metabolism. PPARα regulates the expression of CPT1A, which is involved in β-oxidation (Bechmann et al., 2012). Our results showed that the expression of CPT1A and PPARα was significantly reduced in the liver of cKO F1 mice, indicating the impairment of lipid β-oxidation in cKO F1 mice. This might account for the elevated levels of liver triglycerides, increased lipid droplet deposition, and the decrease in serum triglycerides and VLDL in cKO F1 mice. These aforementioned results indicate changes in the liver lipid metabolism of cKO F1 generation, and suggest that the abnormal fatty acid oxidation pathway may lead to an increase in triglyceride accumulation in the liver. Although cKO F1 mice did not show significant obesity or hyperglycemia, their glucose and lipid metabolism were still dysfunctional, suggesting that cKO F1 mice were at a greater risk of metabolic syndrome than control F1 mice. The overall idea of our research was shown in Figure 9.

In our research, we found that liver triglyceride accumulation was significantly increased in cKO F1 female mice at 9-weeks of age. However, GTT was notably decreased in cKO F1 mice at 9-weeks of age, suggesting that other tissues or cells might be involved in regulating blood glucose, thereby improving glucose tolerance. In addition to the liver, the pancreas also plays a major role in glucose homeostasis. Glucose primarily provides energy by entering cells for oxidation through glucose transporters on the cell surface. GLUT2, one of the important glucose transporters, is found both in the liver and pancreatic β cells (Tirone and Brunicardi, 2001). Lin et al. reported that developmental exposure to di (2-ethylhexyl) phthalate (DEHP) resulted in pancreatic dysfunction and led to whole body glucometabolic abnormalities in rats (Lin et al., 2011). Similarly, Rajesh et al. found that gestational exposure to DEHP caused pancreatic β-cell dysfunction and whole body glucometabolic abnormalities in the F1 offspring due to the down regulation in the expression of critical genes (Rajesh and Balasubramanian, 2015). Niwano et al. suggested that blood glucose levels showed significant change during oral GTT following partial pancreatectomy of different resection types in patients (Niwano et al., 2021), indicating the importance of pancreas in blood glucose regulation. Moreover, other glucose transporters such as GLUT 3 and GLUT 4 are located in neurons, intestine, testicles, kidneys, muscles, and adipose tissues (Tirone and Brunicardi, 2001). Based on the findings of the aforementioned studies, we hypothesized that there may be other tissues involved in the regulation of blood glucose, thereby showing an improvement in GTT but accumulation of triglycerides in the liver. However, the specific mechanism of regulation needs further analysis.

The liver plays a central role in lipid metabolism and secretes VLDL. VLDL secretion requires simultaneous secretion of triglycerides (Rui, 2014). Due to impaired hepatic lipid β-oxidation in cKO F1 mice, the utilization of fatty acids was reduced, and the extracellular release of triglycerides and VLDL also decreased, resulting in lower serum triglyceride and VLDL levels. The significant increase in hepatic triglyceride level in cKO F1 female mice at 9-weeks of age might be caused by decreased oxidative decomposition or increased hepatic triglyceride synthesis. Triglyceride is decomposed and supplied with energy by β-oxidation. The expression of CPT1A and PPARα in liver of cKO female F1 mice at 9-weeks of age was significantly down-regulated, indicating that the liver lipid oxidation capacity was weakened. In addition, if triglyceride synthesis in hepatocytes is inhibited, free fatty acids will accumulate in the liver, leading to the induction of fatty acid oxidation system, increasing hepatic oxidative stress and liver injury (Choi and Diehl, 2008). The level of MDA, the product of lipid peroxidation, was significantly increased in the liver of 9-week-old cKO female F1 mice, indicating oxidative stress in the liver. Therefore, the level of liver triglyceride in mice was significantly increased, which might be mainly due to the decrease of liver triglyceride decomposition. Meanwhile, the inhibition of triglyceride synthesis led to enhanced oxidative stress and increased MDA in the liver. The enhanced glucose tolerance of 9-week-old cKO female F1 mice might be related to the involvement of pancreas and other tissues in blood glucose regulation.

Studies have reported that hormone receptor genes responsible for regulating the actions of estrogen and progesterone, are under epigenetic control in the endometrial tissue (Munro et al., 2010). Metabolic syndrome is mainly characterized by obesity, dyslipidemia, hypertension, insulin resistance, proinflammatory states, *etc.* Affected patients have an increased risk of cardiovascular disease and type 2 diabetes (Grundy, 2008). Hormone plays a vital role in the pathogenesis of metabolic syndrome, its occurrence, and development. Studies conducted on transgenic mice (ArKO mice) show that the inactivation of aromatase enzyme which is essential for estrogen synthesis, demonstrated the importance of estrogen in glucose homeostasis and obesity (Jones et al., 2000). Furthermore, in our previous study, we measured serum estrogen and progesterone levels in control and cKO F0 female mice and found no significant difference between these two groups, indicating that endometrium *DNMT3B* deficiency may not affect sex hormone production (Long et al., 2021).

Epigenetic alterations determine adaptation to changing environmental conditions. Epigenetic modifications provide a possible link between the environment and altered expression of genes that may contribute to disease phenotypes. If epigenetic modifications interfere with gene expression or function, this may lead to a variety of diseases (Jirtle and Skinner, 2007; Machnik and Oleksiewicz, 2020). This depends on the genomic targets and methylation state of different target regions (Jirtle and Skinner, 2007). Changes mediated by maternal stress in the fetal epigenome are responsible for developmental programming and differential adaptive responses in the fetus which may result in phenotypic changes and disorders in adult offspring (Goyal et al., 2019). Suboptimal maternal uterine circumstances, such as the uterus at advanced maternal age, affect the growth and development of offspring (Salem Yaniv et al., 2011). If epigenetic changes occur at critical developmental stages, the epigenome of the germ line may be permanently altered. Additionally, these changes may be passed down through several generations (Bommarito et al., 2017). When epigenetic changes occur in pregnant females (F0 generation), their F1 and F2 generations are directly exposed in the uterus, which may result in intergenerational epigenetic inheritance (Heard and Martienssen, 2014). Studies have reported that, low dose of estradiol-17β exposure in F0 generation sows during pregnancy and found hypomethylation exhibited in specific gene regions in the F0 endometrium, liver and corpus luteum and F1 embryo, but hypermethylation in liver of adult F1 generation (van der Weijden et al., 2018). Maternal uterine exposure to endocrine disruptors and toxic metals affects fetal epigenetic reprogramming (Bommarito et al., 2017). Therefore, we hypothesized that *DNMT3B* deletion in the F0 endometrium caused alterations in the methylation status of certain CpG sites involved in hepatic metabolism related genes of F1 generation, thereby affecting the metabolic ability of F1 generation. However, the mechanisms involved require elucidation.

Sex plays an important role in the pathogenesis of metabolic diseases (Tramunt et al., 2020). Understanding sex differences in metabolism is crucial for the prevention, diagnosis and treatment of metabolic diseases (Link and Reue, 2017). Due to the effects of sex hormone-dependent and independent sex differences on fat distribution, hepatic gluconeogenesis and glycogenolysis, and glucose uptake in skeletal muscle, men may be more prone to elevated fasting glucose, whereas women may be more prone to impaired glucose tolerance (Nuutila et al., 1995; Unwin et al., 2002; Shi et al., 2009). Some epidemiological evidence suggested that diabetes is more prevalent in men, but women with ovarian insufficiency or early menopause are also associated with an increased risk of type 2 diabetes, possibly due to the effect of endogenous estrogen (Mauvais-Jarvis et al., 2013; Anagnostis et al., 2019; Tramunt et al., 2020). Treatment with estradiol or testosterone reduced lipid accumulation in the liver of rats (Zhang et al., 2013). However, sex differences in metabolism, and the corresponding differences in disease risk, cannot be attributed solely to the effects of estrogen or any other sex-related hormone. After removing the gonads of mice, both XX chromosome mice and XY chromosome mice fed a high-fat diet showed obvious deposition of lipid in the liver (Chen et al., 2012). Differences in sensitivity to metabolic hormones and adipokines, such as leptin and insulin, may underlie some of the sex differences in the prevalence of type 2 diabetes and obesity, and interestingly, these hormones and adipokines are often altered by the nutritional environment of early life (Dearden et al., 2018). In this study, significant effects of sex on glucose tolerance and hepatic lipid metabolism were observed. These differences may be related to sex hormones, sex chromosomes or the reprogramming of metabolic development. Knowledge of sex differences in metabolism is important for the development of therapies for specific metabolic pathways and, in the future, for the prevention of risk factors for metabolic diseases.

In conclusion, we demonstrated the effect of maternal endometrial *DNMT3B* deficiency on hepatic metabolism in F1 generation. Though the F1 generation exhibited no overt signs of metabolic diseases, their metabolic function was still impaired, indicating high risk of metabolic diseases. Whether maternal endometrial *DNMT3B* knockout has long-term effects on F1 generation, such as whether the hepatic metabolic function of cKO F1 mice is age-dependent and whether older cKO F1 mice will develop metabolic diseases remain to be further explored. Further research is also required to elucidate the mechanism of the altered maternal uterine methylation status on the metabolism of offspring.

## Data Availability

The original contributions presented in the study are included in the article/Supplementary Material, further inquiries can be directed to the corresponding authors.
